# Developing machine learning models for predicting cardiovascular disease survival based on heavy metal serum and urine levels

**DOI:** 10.3389/fpubh.2025.1582779

**Published:** 2025-05-21

**Authors:** Hui Jin, Ling Zhang, Yan Sun, Ya Xu, Man Luo

**Affiliations:** ^1^Mental Health Center, West China Hospital, Sichuan University, Chengdu, Sichuan, China; ^2^Huai’an No. 3 People’s Hospital, Huaian Second Clinical College, Xuzhou Medical University, Huai’an, Jiangsu, China; ^3^Nanjing Jiangbei Hospital, Affiliated Nanjing Jiangbei Hospital of Xinglin College, Nantong University, Nanjing, Jiangsu, China; ^4^Huai’an TCM Hospital Affiliated to Nanjing University of Chinese Medicine, Huai'an, Jiangsu, China

**Keywords:** interpretable machine learning, heavy metals, cardiovascular disease, mortality, SHAP

## Abstract

**Background:**

Environmental exposure to heavy metals, such as arsenic, cadmium, and lead, is a known risk factor for cardiovascular diseases.

**Objective:**

We aim to examine the associations between heavy metal exposure and the mortality of patients with cardiovascular diseases.

**Methods:**

We analyzed data from the NHANES 2003–2018, including urine and blood metal concentrations from 4,924 participants. Five machine learning models—CoxPHSurvival, FastKernelSurvivalSVM, GradientBoostingSurvival, RandomSurvivalForest, and ExtraSurvivalTrees—were used to predict cardiovascular mortality. Model performance was assessed with the concordance index (C-index), integrated Brier score, time-dependent AUC, and calibration curves. SHAP analysis was conducted using a reduced background dataset created via K-means clustering.

**Results:**

GradientBoostingSurvival (GBS) showed the best performance for hypertension (C-index: 0.780, mean AUC: 0.798). RandomSurvivalForest (RSF) was the top model for coronary heart disease (C-index: 0.592, mean AUC: 0.626) and myocardial infarction (C-index: 0.705, mean AUC: 0.743), while CoxPHSurvival excelled for heart failure (C-index: 0.642, mean AUC: 0.672) and stroke (C-index: 0.658, mean AUC: 0.691). ExtraSurvivalTrees performed best in angina (C-index: 0.652, mean AUC: 0.669). Calibration curves confirmed the models’ accuracy. SHAP analysis identified age as the most influential factor, with heavy metals like lead, cadmium, and thallium significantly contributing to risk. A user-friendly web calculator was developed for individualized survival predictions.

**Conclusion:**

Machine learning models, including GradientBoostingSurvival, RandomSurvivalForest, CoxPHSurvival, and ExtraSurvivalTrees, demonstrated strong performance in predicting mortality risk for various cardiovascular diseases. Key metals were identified as significant risk factors in cardiovascular risk assessment.

## Introduction

Cardiovascular diseases remain the leading cause of mortality worldwide, accounting for approximately 19 million deaths in 2021, with ischemic heart disease and stroke collectively responsible for 23% of global deaths (13 and 10% respectively), representing most of the cardiovascular-related mortality (1). Therefore, the precise prediction and effective management of cardiovascular risk in this population are critically important (2, 3). The rapid advancements in machine learning (ML) have sparked optimism for a more personalized, efficient, and effective strategy in managing cardiovascular disease and its associated cardiovascular complications (4).

Heavy metal pollution, including toxic metals like arsenic, cadmium, lead, and mercury, as well as essential trace metals such as chromium, copper, and zinc, poses significant health risks due to increased industrialization and anthropogenic activities (5, 6). Environmental exposure to heavy metals is a modifiable risk factor for CVDs (7–10). Evidence over the past two decades has shown that heavy metals contribute to CVD risk through their lasting effects on the cardiovascular system, including hypertension (11), atherosclerosis (12) and stroke (13). Furthermore, imbalances in essential trace metals, such as high copper or low magnesium and zinc levels, are associated with increased cardiovascular mortality (14). Several studies have developed machine learning models to predict CVDs using various factors (15–18). For example, Ambale-Venkatesh et al. employed random survival forests to predict six cardiovascular outcomes in an asymptomatic population, showing enhanced accuracy compared to traditional risk scores (19). However, no study has yet developed machine learning models to predict the survival of CVDs patients, including those with hypertension, coronary heart disease, heart failure, myocardial infarction, and stroke.

In this study, we used data from the National Health and Nutrition Examination Survey (NHANES, 2003–2018) to examine the associations between heavy metal exposure and the mortality of patients with CVDs. We developed five machine learning survival models to predict the mortality of CVD patients based on heavy metal exposure and compared their performance. Additionally, we employed an advanced ML technique, SHapley Additive exPlanations (SHAP), to evaluate the contribution of each heavy metal to the survival models, thereby enhancing the potential for early intervention. Furthermore, we developed a user-friendly web calculator that allows clinicians and patients to compute individualized survival predictions for different cardiovascular diseases, offering a practical tool for risk assessment and informed decision-making. Our findings have significant public health implications, as they highlight the importance of reducing exposure to heavy metals and provide a valuable tool for identifying high-risk individuals who may benefit from targeted interventions and increased monitoring. By addressing the environmental factors contributing to CVD mortality, we can work towards reducing the global burden of these diseases and improving overall population health.

## Methods

### Population

This study utilized data from the National Health and Nutrition Examination Survey (NHANES), an ongoing, nationally representative survey designed by the U.S. Centers for Disease Control and Prevention (CDC) to monitor the health and nutritional status of the U.S. population. The survey procedures were approved by the Institutional Review Board of the National Center for Health Statistics (NCHS). Additional information about NHANES is accessible on its official website (National Center for Health Statistics, https://www.cdc.gov/nchs/nhanes/index.htm). For this research, we extracted data related to cardiovascular diseases, including hypertension, heart failure, coronary heart disease, myocardial infarction, and stroke, from the NHANES cycles conducted between 2003 and 2018. The total number of participants across these cycles was 80,312. The dataset contained detailed measurements of urine and blood metal concentrations, along with various covariates. A total of 66,264 individuals were missing data on metal concentrations in blood or urine, which are crucial variables for this study. Additionally, missing data was observed for essential diagnosis variables, with 28 individuals missing hypertension data, 38 missing heart failure data, 57 missing coronary heart disease data, 20 missing myocardial infarction data, 38 missing angina data, and 16 missing stroke data. Furthermore, 160 individuals had missing BMI data, which is an important covariate in the analysis of cardiovascular disease risk factors. The KNN imputation method was applied to address the missing BMI data (less than 20% missing data), ensuring a complete dataset for analysis. The final cohort for analysis included participants with complete datasets, yielding a total sample size of 4,924. A comprehensive flowchart illustrating the participant selection process is provided in Supplementary Figure S1. Our primary outcome of interest was all-cause mortality.

### Data collection

Demographic information for participants was obtained from the NHANES questionnaire data. The collected characteristics included gender (male and female), age (in years), race/ethnicity (Mexican American, Other Hispanic, Non-Hispanic White, Non-Hispanic Black, and other races), educational level (high school or lower, more than high school), weight (kg), height (cm), and body mass index (BMI, kg/m^2^). Cardiovascular disease status was determined through self-reported physician diagnoses using a standardized medical conditions questionnaire during individual interviews. Participants were asked, “Have you ever been told you have congestive heart failure?,” “Have you ever been told you have coronary heart disease?,” “Have you ever been told you had a heart attack?,” “Have you ever been told you had a angina?,” “Have you ever been told you had a stroke?,” and “Have you ever been told you have high blood pressure?” with responses recorded as either “yes” or “no”. Blood and urine samples were collected by trained personnel, stored at −20°C, and subsequently sent to the Laboratory Science Division of the National Center for Environmental Health at the CDC for analysis. Detailed procedures for blood and urine sample collection and processing for cadmium concentration measurements are outlined in the NHANES Laboratory Technologists Procedures Manual.^1^ For our analysis, we included 21 heavy metals measured in both urine and blood, with all relevant details presented in Table 1. The mortality status of the follow-up population was determined using the NHANES public-use linked mortality file, updated as of December 31, 2019, and matched to the National Death Index (NDI) through a probability-based algorithm by the NCHS. Cause-specific mortality was classified based on the International Statistical Classification of Diseases, 10th Revision (ICD-10), including heart diseases (codes 054–064), malignant neoplasms (codes 019–043), and other causes (code 010).

**Table 1:** Demographic characteristics of patients with cardiovascular disease.

Characteristics	Overall (*N* = 4,924)
Gender, *n* (%)	
Male	2457 (49.9%)
Female	2467 (50.1%)
Age, years	60.4 (14.9)
Race, *n* (%)	
Mexican American	610 (12.4%)
Other Hispanic	380 (7.7%)
Non-Hispanic White	2267 (46.0%)
Non-Hispanic Black	1251 (25.4%)
Other	416 (8.4%)
Education, *n* (%)	
High school or lower	2631 (53.4%)
More than high school	2293 (46.6%)
Weight, kg	85.9 (22.6)
Height, cm	167 (10.3)
BMI, kg/m^2^	30.8 (7.12)
Hypertension, *n* (%)	4592 (93.3%)
Heart failure, *n* (%)	373 (7.6%)
Coronary heart disease, *n* (%)	485 (9.8%)
Angina, *n* (%)	315 (6.4%)
Myocardial infarction, *n* (%)	534 (10.8%)
Stroke, *n* (%)	491 (10.0%)
All-cause mortality, *n* (%)	1022 (20.8%)
Cardiovascular mortality, *n* (%)	356 (7.2%)
Follow-up time, months	91.1 (52.6)

### Data preprocessing and variable selection

In this study, both categorical variables (gender, race/ethnicity, education level) and numerical variables (age, BMI, urine total arsenic, arsenic acid, arsenous acid, arsenobetaine, arsenocholine, dimethylarsinic acid, monomethylarsonic acid, blood and urine concentrations of lead, cadmium, mercury [including blood inorganic mercury], and urine concentrations of barium, cobalt, cesium, molybdenum, antimony, thallium, tungsten) were included in the machine learning models. To standardize the numerical data, MinMaxScaler was performed, rescaling all numerical values to a range of 0 to 10 to ensure uniformity in variable scales for model fitting. The scaled data were then converted to a float format to facilitate compatibility with the survival models. These standardized numerical variables were combined with the categorical variables into a single data frame for subsequent analysis. The target variables for survival analysis, namely “Survival months” (as the time variable) and “Survival status” (as the event indicator), were extracted from this data frame. “Survival status” was encoded as a Boolean variable, with True indicating an event occurrence and False indicating censoring. In order to ensure the reliability and accuracy of our analysis, we performed a thorough data preprocessing and variable selection process. We conducted a multicollinearity check on all variables to identify and remove those with high collinearity, which could potentially distort the results of our machine learning models. Specifically, we calculated the Variance Inflation Factor (VIF) for each variable and excluded all variables with a VIF exceeding the threshold of 10, as shown in Supplementary Table S1.

### Training machine learning models

**Figure 1:**
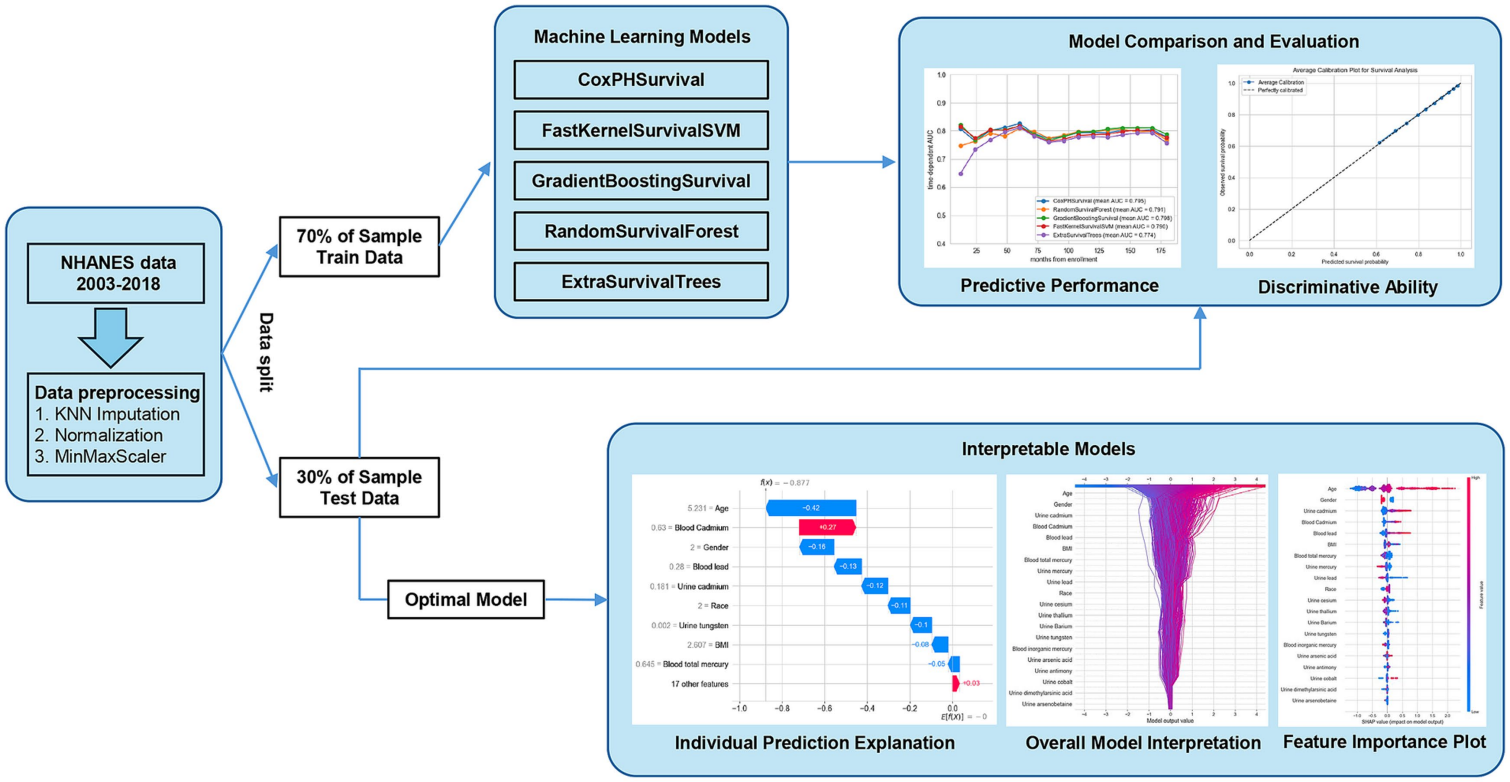
Machine learning workflow for survival analysis.

Machine learning workflow for survival analysis was shown in Figure 1. The dataset was split into training and testing sets in a 7:3 ratio to facilitate model training and evaluation. The survival target was structured as an array containing both event status and survival duration, formatted to meet the input requirements of the survival analysis models. Using the training set, five machine learning algorithms (CoxPHSurvival, FastKernelSurvivalSVM, GradientBoostingSurvival, RandomSurvivalForest, and ExtraSurvivalTrees) were constructed to predict mortality among patients with cardiovascular conditions, including hypertension, coronary heart disease, heart failure, myocardial infarction, angina, and stroke (Figure 1). These models were implemented using the “NumPy”, “Pandas”, and “scikit-survival” Python packages on a Windows operating system. Detailed information on each algorithm is available in the Supplementary material and on the official scikit-survival website (scikit-survival 0.23.0, https://scikit-survival.readthedocs.io/en/stable/). The hyperparameter grid search for each model was performed to find the best model. Various machine learning models are trained and evaluated for predictive performance.

### Performance metrics

The performance of the survival models was evaluated using the concordance index (C-index), integrated Brier score, time-dependent area under the curve (td-AUC), and calibration curves. The C-index (Harrell’s C-index) measured the models’ discriminatory power, while the Integrated Brier score (IBS) assessed prediction accuracy over time. We used the “integrated_brier_score” function to evaluate the prediction performance of the model (20). Specifically, we set the time interval from the 12th month to the 180th month (one time point every 12 months) and calculated the IBS in this interval to measure the prediction accuracy of the model at different follow-up time points. IBS provides an assessment of the overall calibration ability of the model by calculating the weighted integral of the Brier score (i.e., the mean square error between the predicted probability and the actual survival status) over the entire time window. Lower IBS values indicate that the deviation between the model’s survival probability prediction and the observed value is smaller and the prediction performance is better. Therefore, in this study, we used the integrated_brier_score function provided by sksurv.metrics to perform integral calculations for the 12–180 month interval and used Bootstrap sampling (repeated 1,000 times) to estimate the 95% confidence interval to ensure the robustness of the estimation results. Time-dependent AUCs provided insights into the models’ ability to distinguish between events at various time points. We used the “cumulative_dynamic_auc” function provided by the sksurv.metrics module, which uses the cumulative/dynamic method based on inverse probability weighting (IPCW) to calculate the AUC over time. Calibration curves were used to compare predicted and observed survival probabilities, with 10-fold cross-validation ensuring robust estimates. These performance metrics, particularly C-index and td-AUC, were the primary criteria for model selection, while IBS and calibration curves were considered as supplementary factors to assess overall model performance.

### SHAP analysis for model interpretation

To identify and interpret the most influential features in predicting cardiovascular outcomes, we used SHapley Additive exPlanations (SHAP) with the Extra Trees model. Given the computational intensity of SHAP, we first reduced the background dataset using K-means clustering on the training data to generate 50 cluster centers as the background data for SHAP analysis. A KernelExplainer was then created using this reduced background and the survival models. SHAP values for the test dataset were calculated to quantify each feature’s contribution to the model’s predictions. Feature importance was assessed by calculating the mean absolute SHAP values across all test samples, and the top 20 most important features were identified. To visualize the results, a SHAP decision plot was generated to show the cumulative impact of the top 20 features on model predictions, highlighting their positive or negative contributions. Additionally, a SHAP summary plot was created to depict the distribution and influence of these top features across the test set, offering insights into their significance in the model’s predictions.

### Web-based calculator development

To translate our findings into a practical tool, we developed a web-based calculator using the Gradio library in Python. Gradio allows for the rapid creation of interactive web interfaces for machine learning models. The calculator’s user interface includes input fields for the key predictors identified in our analysis for different CVD. Upon clicking a “Make Predict” button, the user-provided inputs are preprocessed (including standardization of numerical features using the same StandardScaler used during model training), fed into the trained optimal model, and the predicted survival data. The source code and deployment details are available on Hugging Face Spaces (Hypertension: https://huggingface.co/spaces/MLML202512/Hypertension; CHD: https://huggingface.co/spaces/MLML202512/CHD; HF: https://huggingface.co/spaces/MLML202512/HF; MI: https://huggingface.co/spaces/MLML202512/MI; Stroke: https://huggingface.co/spaces/MLML202512/Stroke); Angina: https://huggingface.co/spaces/MLML202512/angina.

### Statistical analysis

Categorical variables were summarized as frequencies and percentages, while continuous variables were reported as medians and standard deviations. Differences across the NHANES cycles were evaluated using either the Chi-squared test or the Kruskal-Wallis H test. A two-sided *p*-value of less than 0.05 was considered statistically significant. All statistical analyses were conducted using Python (version 3.12.0) and R software (version 4.4.0).

## Results

### Characteristics of the study population

Table 1 presented the demographic and clinical characteristics of the 4,924 participants with cardiovascular disease. The cohort had a nearly equal gender distribution (49.9% male and 50.1% female) with a mean age of 60.4 ± 14.9 years. Most participants were Non-Hispanic White (46.0%), followed by Non-Hispanic Black (25.4%), Mexican American (12.4%), Other Hispanic (7.7%), and Other (8.4%). In terms of education, 53.4% had a high school education or lower. The average weight was 85.9 ± 22.6 kg, and the mean BMI was 30.8 ± 7.12 kg/m^2^. Hypertension was prevalent in 93.3% of the participants, while heart failure, coronary heart disease, myocardial infarction, angina, and stroke were present in 7.6, 9.8, 10.8, 6.4, and 10.0% of the cohort, respectively. All-cause mortality occurred in 20.6% of the participants, with cardiovascular mortality accounting for 7.2%. The mean follow-up period was 91.1 ± 52.6 months.

### Concentrations of heavy metals

As shown in Table 2, we presented the concentrations of various heavy metals in urine and blood across different NHANES survey cycles from 2003 to 2018. Significant changes over time were observed in several heavy metals. In urine, the concentrations of total arsenic, arsenous acid, arsenic acid, dimethylarsinic acid, monomethylarsonic acid, barium, cadmium, lead, antimony, and tungsten showed significant trends (all *p*-values < 0.05). Similarly, significant trends were noted in blood concentrations of cadmium and lead across the survey cycles (all *p*-values < 0.05). These changes highlight potential shifts in exposure to heavy metals over the 16-year period.

**Table 2:** Demographic characteristics of heavy metals across each cycle of the NHANES database (2003–2018).

Heavy metal	Cycles of NHANES									*p*-value
	2003–2004	2005–2006	2007–2008	2009–2010	2011–2013	2013–2014	2015–2016	2017–2018	Total	
	(*N* = 568)	(*N* = 465)	(*N* = 625)	(*N* = 703)	(*N* = 583)	(*N* = 666)	(*N* = 639)	(*N* = 675)	(*N* = 4,924)	
Urine										
Total Arsenic (μg/L)	20.9 (65.3)	26.5 (72.7)	14.8 (29.6)	21.9 (52.2)	20.5 (60.8)	18.7 (51.8)	18.2 (44.8)	17.8 (39.2)	19.7 (52.6)	<0.001
Arsenic acid (μg/L)	0.74 (0.17)	0.78 (0.65)	0.71 (0.04)	0.72 (0.07)	0.63 (0.12)	0.57 (0.13)	0.58 (0.10)	0.60 (0.17)	0.66 (0.24)	<0.001
Arsenous acid (μg/L)	0.83 (0.23)	0.89 (0.31)	0.86 (0.11)	0.87 (0.21)	0.46 (0.28)	0.44 (0.44)	0.31 (0.37)	0.24 (0.34)	0.60 (0.40)	<0.001
Arsenobetaine (μg/L)	11.6 (54.2)	15.9 (59.1)	6.1 (22.4)	10.8 (38.1)	12.3 (53.3)	12.0 (50.2)	11.8 (41.8)	10.0 (30.8)	11.1 (44.5)	<0.001
Arsenocholine (μg/L)	0.43 (0.24)	0.45 (0.23)	0.43 (0.08)	0.47 (0.61)	0.25 (0.35)	0.16 (0.59)	0.18 (0.46)	0.20 (0.10)	0.31 (0.55)	<0.001
Dimethylarsinic acid (μg/L)	4.99 (5.53)	5.86 (9.43)	5.05 (5.23)	5.69 (9.75)	6.20 (8.58)	5.17 (6.63)	4.97 (5.50)	5.00 (6.77)	5.34 (7.35)	0.015
Monomethylarsonic acid (ng/mL)	0.90 (0.76)	0.98 (1.37)	0.89 (0.58)	1.11 (6.66)	0.84 (0.49)	0.60 (0.74)	0.49 (0.49)	0.45 (0.51)	0.77 (2.61)	<0.001
Lead (ng/mL)	1.01 (1.14)	0.97 (0.88)	0.79 (0.85)	0.80 (0.91)	0.65 (0.86)	0.51 (0.48)	0.57 (0.90)	0.50 (0.50)	0.71 (0.85)	<0.001
Mercury (ng/mL)	0.67 (0.95)	0.82 (1.04)	0.72 (1.04)	0.63 (0.77)	0.71 (2.35)	0.58 (1.91)	0.36 (1.21)	0.36 (0.86)	0.59 (1.38)	<0.001
Barium (ng/mL)	1.95 (3.94)	1.79 (2.18)	2.02 (5.15)	1.87 (2.41)	1.66 (4.18)	1.58 (3.19)	1.58 (2.19)	1.46 (2.19)	1.73 (3.34)	<0.001
Cadmium (ng/mL)	0.56 (0.60)	0.51 (0.51)	0.48 (0.51)	0.46 (0.49)	0.48 (0.61)	0.41 (0.47)	0.40 (0.39)	0.41 (0.50)	0.45 (0.51)	<0.001
Cobalt (ng/mL)	0.46 (1.16)	0.68 (2.40)	0.46 (0.68)	0.60 (1.81)	0.48 (1.37)	0.61 (1.76)	0.59 (1.08)	0.63 (1.39)	0.56 (1.51)	<0.001
Cesium (ng/mL)	6.15 (13.8)	5.50 (3.65)	5.11 (4.62)	4.73 (3.09)	4.92 (3.16)	4.92 (3.28)	5.08 (3.73)	5.12 (3.38)	5.16 (5.79)	0.069
Molybdenum (ng/mL)	55.9 (72.9)	54.1 (49.4)	57.7 (55.3)	53.1 (47.1)	53.7 (50.5)	45.3 (39.3)	50.8 (46.3)	45.6 (39.7)	51.8 (50.7)	<0.001
Antimony (ng/mL)	0.10 (0.09)	0.10 (0.11)	0.08 (0.08)	0.07 (0.10)	0.07 (0.09)	0.07 (0.19)	0.08 (0.17)	0.07 (0.13)	0.08 (0.13)	<0.001
Thallium (ng/mL)	0.17 (0.14)	0.17 (0.12)	0.17 (0.18)	0.17 (0.15)	0.19 (0.14)	0.17 (0.13)	0.19 (0.32)	0.18 (0.13)	0.18 (0.18)	0.003
Tungsten (ng/mL)	0.10 (0.12)	0.14 (0.32)	0.17 (0.41)	0.10 (0.13)	0.12 (0.22)	0.10 (0.21)	0.10 (0.15)	0.10 (0.21)	0.11 (0.24)	<0.001
Blood										
Lead (μg/dL)	2.40 (1.66)	2.31 (1.64)	2.06 (1.50)	1.93 (1.62)	1.69 (1.82)	1.51 (1.41)	1.49 (1.39)	1.42 (1.17)	1.83 (1.57)	<0.001
Cadmium (μg/L)	0.60 (0.65)	0.60 (0.70)	0.57 (0.59)	0.54 (0.51)	0.55 (0.56)	0.56 (0.60)	0.53 (0.57)	0.56 (0.72)	0.56 (0.61)	<0.001
Total mercury (μg/L)	1.33 (1.42)	1.64 (2.02)	1.37 (1.73)	1.69 (2.79)	1.51 (1.87)	1.56 (2.58)	1.45 (2.10)	1.43 (2.17)	1.50 (2.16)	<0.001
Inorganic mercury (μg/L)	0.37 (0.18)	0.34 (0.17)	0.32 (0.17)	0.30 (0.24)	0.27 (0.24)	0.29 (0.53)	0.25 (0.18)	0.25 (0.81)	0.29 (0.40)	<0.001

### Evaluation and comparison of models

**Figure 2:**
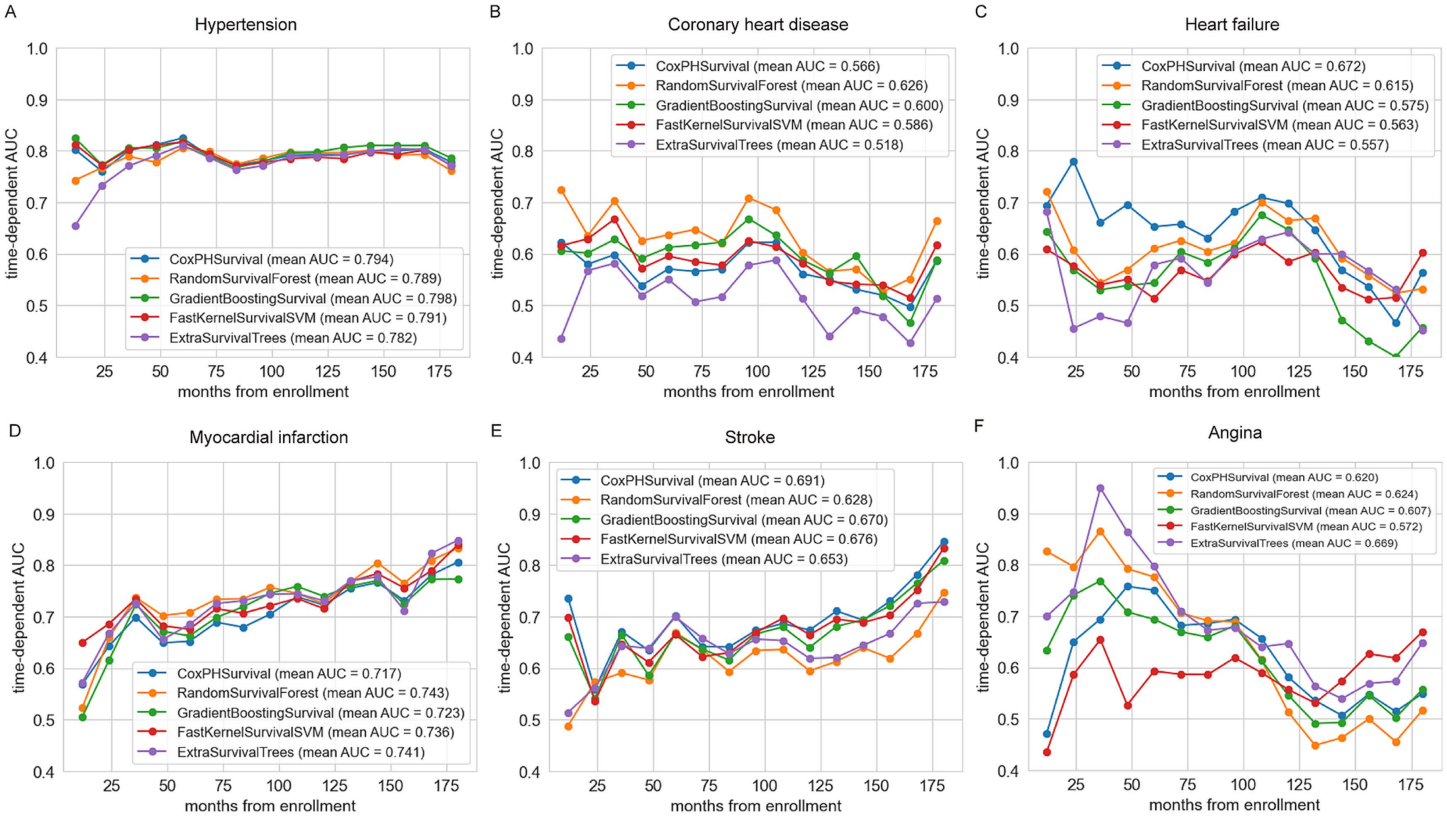
Summary of time-dependent area under the curve (td-AUC) performance for five survival models predicting cardiovascular disease mortality. **(A)** Hypertension; **(B)** Coronary heart disease; **(C)** Heart failure; **(D)** Myocardial infarction; **(E)** Stroke; **(F)** Angina.

**Figure 3:**
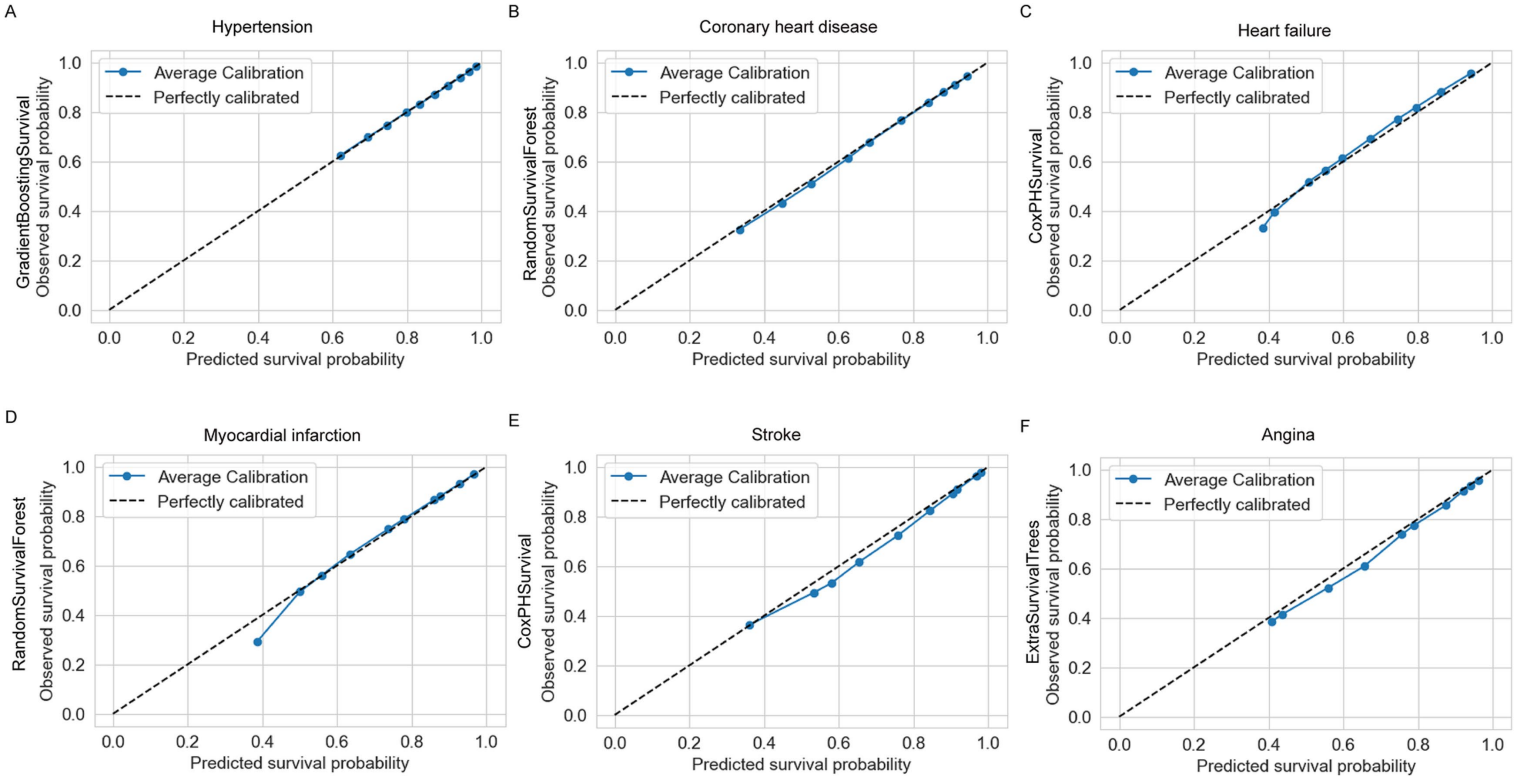
Calibration plots of the best-performing models for each cardiovascular disease. **(A)** Hypertension: GradientBoostingSurvival, **(B)** coronary heart disease: RandomSurvivalForest, **(C)** heart failure: CoxPHSurvival, **(D)** myocardial infarction: RandomSurvivalForest, **(E)** stroke: CoxPHSurvival, **(F)** Angina: ExtraSurvivalTrees.

The performance of each model was evaluated using the concordance index (C-index), time-dependent AUC, and calibration curves. The C-index measured the discriminative ability of each model, where higher values indicate better differentiation between patients with varying survival times (Table 3). Time-dependent AUC was utilized to graphically demonstrate the models’ predictive accuracy over time, with higher mean AUC values indicating better performance (Figure 2). Calibration curves assessed the alignment between predicted and observed survival probabilities, ensuring well-calibrated predictions (Figure 3; Supplementary Figures S2–S7).

**Table 3:** Discriminative ability and calibration of each model in predicting all-cause mortality among cardiovascular disease patients.

Diseases	c-index (95% CI)	Brier score (95% CI)
Hypertension		
CoxPHSurvival	0.778 (0.749-0.806)	0.110 (0.099-0.121)
FastKernelSurvivalSVM	0.776 (0.746-0.805)	/
GradientBoostingSurvival	0.780 (0.748-0.809)	0.111 (0.100-0.124)
RandomSurvivalForest	0.779 (0.749-0.808)	0.111 (0.100-0.123)
ExtraSurvivalTrees	0.780 (0.750-0.809)	0.112 (0.100-0.123)
Coronary heart disease		
CoxPHSurvival	0.549 (0.473-0.628)	0.208 (0.174-0.248)
FastKernelSurvivalSVM	0.570 (0.481-0.655)	/
GradientBoostingSurvival	0.580 (0.487-0.665)	0.202 (0.170-0.241)
RandomSurvivalForest	0.592 (0.494-0.688)	0.199 (0.166-0.236)
ExtraSurvivalTrees	0.584 (0.484-0.684)	0.200 (0.167-0.234)
Angina		
CoxPHSurvival	0.642 (0.523-0.752)	0.203 (0.153-0.260)
FastKernelSurvivalSVM	0.616 (0.502-0.735)	/
GradientBoostingSurvival	0.626 (0.517-0.734)	0.202 (0.154-0.255)
RandomSurvivalForest	0.629 (0.519-0.735)	0.198 (0.152-0.252)
ExtraSurvivalTrees	0.652 (0.529-0.772)	0.189 (0.146-0.246)
Heart failure		
CoxPHSurvival	0.642 (0.556-0.720)	0.218 (0.179-0.265)
FastKernelSurvivalSVM	0.605 (0.494-0.711)	/
GradientBoostingSurvival	0.591 (0.488-0.702)	0.217 (0.182-0.258)
RandomSurvivalForest	0.591 (0.492-0.698)	0.215 (0.182-0.253)
ExtraSurvivalTrees	0.587 (0.494-0.694)	0.217 (0.184-0.251)
Myocardial infarction		
CoxPHSurvival	0.681 (0.595-0.761)	0.179 (0.143-0.219)
FastKernelSurvivalSVM	0.694 (0.608-0.775)	/
GradientBoostingSurvival	0.696 (0.610-0.777)	0.174 (0.141-0.212)
RandomSurvivalForest	0.705 (0.614-0.788)	0.168 (0.134-0.209)
ExtraSurvivalTrees	0.705 (0.617-0.788)	0.168 (0.136-0.206)
Stroke		
CoxPHSurvival	0.658 (0.576-0.739)	0.180 (0.141-0.222)
FastKernelSurvivalSVM	0.651 (0.569-0.729)	/
GradientBoostingSurvival	0.643 (0.555-0.725)	0.181 (0.145-0.220)
RandomSurvivalForest	0.636 (0.547-0.722)	0.182 (0.146-0.219)
ExtraSurvivalTrees	0.636 (0.547-0.722)	0.180 (0.147-0.218)

For hypertension, the best model was GradientBoostingSurvival (GBS), with a C-index of 0.780 (95% CI: 0.748–0.809) and the highest mean AUC of 0.798 (Figure 2A). The calibration curve for GBS also indicated good agreement between predicted and actual survival probabilities (Figure 3A; Supplementary Figure S2). For coronary heart disease (CHD), RandomSurvivalForest (RSF) performed best, achieving a C-index of 0.592 (95% CI: 0.494–0.688) and the highest mean AUC of 0.626 (Figure 2B). The RSF calibration curve demonstrated close alignment with the ideal reference line, indicating accurate risk prediction (Figure 3B; Supplementary Figure S3). For heart failure, CoxPHSurvival was identified as the top-performing model, with a C-index of 0.642 (95% CI: 0.556–0.720) and a mean AUC of 0.672 (Figure 2C). Its calibration curve further confirmed its superior performance, showing a strong fit with observed probabilities (Figure 3C; Supplementary Figure S4). For myocardial infarction, RandomSurvivalForest (RSF) once again emerged as the optimal model, achieving the highest C-index of 0.705 (95% CI: 0.614–0.788) and a mean AUC of 0.743 (Figure 2D). The model’s calibration curve for myocardial infarction indicated generally reliable prediction accuracy, with the average calibration line closely following the perfectly calibrated reference line for most predicted survival probabilities between 0.5 and 1.0 (Figure 3D; Supplementary Figure S5). However, there appears to be some underestimation in the lower probability range (around 0.4), where the observed survival probability was higher than predicted. For stroke, CoxPHSurvival stood out with a C-index of 0.658 (95% CI: 0.576–0.739) and a mean AUC of 0.691 (Figure 2E). Its calibration curve exhibited excellent agreement between the predicted and observed survival probabilities, underscoring its predictive reliability (Figure 3E; Supplementary Figure S6). Finally, for angina, ExtraSurvivalTrees performed best with a C-index of 0.652 (95% CI: 0.529–0.772) and a mean AUC of 0.669 (Figure 2F). The model’s calibration curve verified its excellent fit with observed probabilities, confirming its superior performance (Figure 3F; Supplementary Figure S7).

### Visualization of feature importance

**Figure 4:**
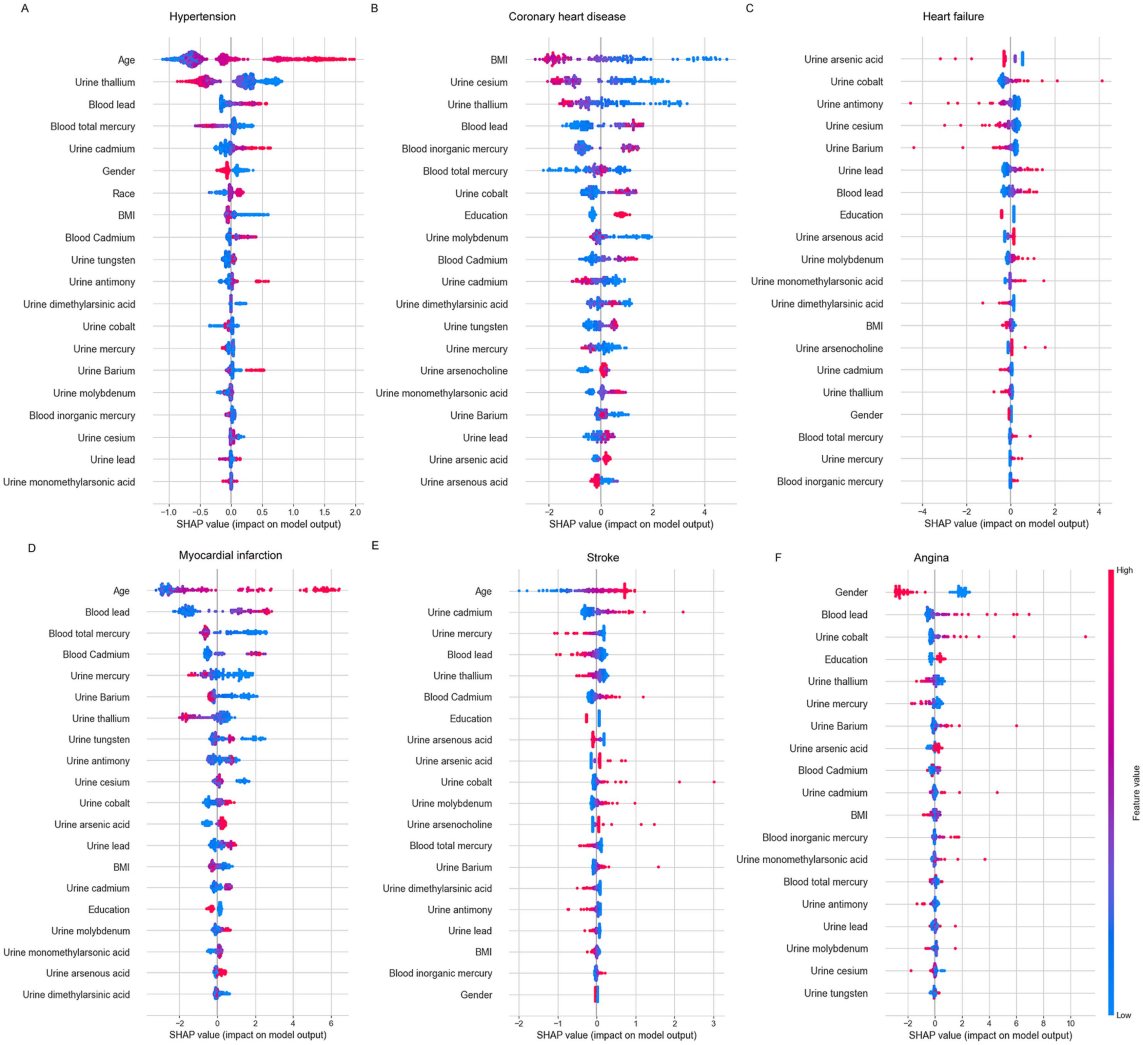
The SHAP summary plots of the best-performing models for each cardiovascular disease. **(A)** Hypertension: GradientBoostingSurvival, **(B)** coronary heart disease: RandomSurvivalForest, **(C)** heart failure: CoxPHSurvival, **(D)** myocardial infarction: RandomSurvivalForest, **(E)** stroke: CoxPHSurvival, **(F)** Angina: ExtraSurvivalTrees.

Figure 4 illustrates the SHAP values for key features influencing the risk of various cardiovascular diseases. Age emerges as the most significant factor across most of conditions, with older age (shown in red) being associated with a higher risk (Figures 4A–E; Supplementary Figures S9A–E). In the case of hypertension (Figure 4A; Supplementary Figure S9A), positive contributions to the model are observed from blood lead, urine cadmium, blood cadmium, urine antimony, and urine barium. Conversely, negative contributions are noted for urine thallium, blood total mercury, male sex, lower BMI, and urine mercury. For coronary heart disease (Figure 4B; Supplementary Figure S9B), factors such as higher education level, blood lead, blood inorganic mercury, urine cobalt, urine arsenocholine, and urine arsenic acid contribute positively to the model. In contrast, lower BMI, urine cesium, urine thallium, blood cadmium, urine molybdenum, and urine arsenous acid exert negative effects. Regarding heart failure (Figure 4C; Supplementary Figure S9C), variables like urine cobalt, urine total arsenic, and urine lead demonstrate minimal influence, as indicated by SHAP values concentrated around zero, reflecting their limited predictive power. For myocardial infarction (Figure 4D; Supplementary Figure S9D), positive contributions are made by blood lead, blood cadmium, and urine arsenic acid, while negative contributions are associated with urine barium, blood total mercury, urine thallium, urine mercury, and urine cesium. In the case of stroke (Figure 4E; Supplementary Figure S9E), positive contributions arise from urine cadmium, urine arsenic acid, urine molybdenum, blood cadmium, urine barium, urine cobalt, and urine arsenocholine, whereas negative contributions are linked to urine mercury, blood lead, urine thallium, urine antimony, urine dimethylarsinic acid, lower education, male sex, blood total mercury, and lower BMI. Lastly, In the case of angina (Figure 4F; Supplementary Figure S9F), positive contributions to the model are observed from blood lead, urine cobalt, urine barium, urine arsenic acid, blood inorganic acid. Conversely, negative contributions are noted for female sex, urine thallium, urine mercury, urine antimony. SHAP waterfall plots presented the profiles of patients at either increased or decreased mortality of cardiovascular diseases and provided promising individualized care planning based on the best model (Supplementary Figures S8A–E).

### Web-based calculator implementation

To enhance the practical application of our research findings, we developed interactive online calculators for predicting various CVDs. These web-based tools were built using the Gradio framework in Python, which facilitates the creation of user-friendly interfaces for machine learning models. Each calculator features intuitive input fields corresponding to the significant predictors identified in our analysis for the respective CVD conditions. All calculators are publicly accessible through Hugging Face Spaces platform (Hypertension: https://huggingface.co/spaces/MLML202512/Hypertension; CHD: https://huggingface.co/spaces/MLML202512/CHD; HF: https://huggingface.co/spaces/MLML202512/HF; MI: https://huggingface.co/spaces/MLML202512/MI; Stroke: https://huggingface.co/spaces/MLML202512/Stroke; Angina: https://huggingface.co/spaces/MLML202512/angina).

## Discussion

In this study, we employed five interpretable machine learning approaches to assess the association between heavy metal exposure and cardiovascular disease mortality among patients in the NHANES database from 2003 to 2018. Each machine learning model demonstrated varying degrees of performance across different cardiovascular diseases. For hypertension, the GBS model outperformed others, achieving a high C-index of 0.780, the highest mean AUC of 0.798, and well-aligned calibration curves. For coronary heart disease, the RSF model was selected due to its highest mean AUC of 0.626 and strong calibration. For heart failure, the CoxPHSurvival model proved to be the most effective, with the highest C-index (0.642), greatest mean AUC (0.672), and accurate calibration. For myocardial infarction, the RSF model demonstrated superior performance, with the highest C-index (0.705), a high mean AUC (0.743), and reliable calibration curves. For stroke, the CoxPHSurvival model was identified as the best, achieving the highest C-index (0.658) and mean AUC (0.691), along with well-fitted calibration curves. Lastly, for angina, the ExtraSurvivalTrees model was identified as the best, achieving the highest C-index (0.652) and mean AUC (0.669), along with well-fitted calibration curves. By integrating these models with SHAP analysis, we provide robust and interpretable predictions for each cardiovascular condition. A user-friendly web calculator was developed for individualized survival predictions. The findings from this research have significant public health implications by enhancing our understanding of how heavy metal exposures contribute to cardiovascular disease burden. Our predictive models and web-based calculators offer practical tools for healthcare providers to incorporate environmental exposure data into cardiovascular risk assessments.

Exposure to environmental factors, particularly heavy metals, is a key and modifiable component of cardiovascular disease (CVD) risk (21). Increasing evidence highlights the significant role that heavy metal exposure plays in the development and progression of CVD (11, 13, 22–24). Machine learning techniques have been increasingly employed in previous research to develop predictive models for various adverse health outcomes related to environmental exposures (25–30). Limited research has explored the association between heavy metals and cardiovascular disease using machine learning methods. Li et al. established RF model to identify associations between heavy metals’ exposure and CHD among US NHANES 2003–2018 participants (25). Li et al. employed nine ML models to establish a predictive model for hypertension utilizing heavy metal exposure data from the NHANES (26). However, the exploration of predictive models specifically linking heavy metal exposure to cardiovascular mortality remains relatively limited. A previous study offered insights into a potential positive association between concentrations of heavy metal mixtures and overall, cardiovascular, and cancer mortality in a large sample of the U.S. general population (31). Building on this research, we developed five different machine learning models to evaluate the association between heavy metal exposure and cardiovascular diseases’ mortality in the U.S. general population.

We observed the significant temporal trends observed in Table 2 for various heavy metal concentrations across different NHANES survey cycles from 2003 to 2018. These temporal trends may be attributed to several factors, including changes in industrial practices, implementation of stricter environmental regulations, shifts in consumer product formulations, and evolving occupational exposure standards. The decline in lead and cadmium levels, in particular, likely reflects the success of targeted regulatory efforts in reducing industrial emissions, phasing out leaded gasoline, and restricting the use of these metals in consumer products. Conversely, changes in other metal concentrations may reflect emerging industrial applications or the identification of previously unrecognized exposure sources. These findings underscore the dynamic nature of environmental exposures and the potential impact of policy interventions on population-level heavy metal burden.

In this study, we utilized five machine learning models, with a particular focus on the Gradient Boosting Survival method, renowned for its rapid computational speed, strong generalization ability, and high predictive performance (32). Advanced techniques, such as KNN for missing value imputation and GridSearchCV for hyperparameter optimization, were also employed to enhance the analysis. Our results demonstrate that these methods significantly improve the prediction of all-cause mortality in patients with cardiovascular disease. Furthermore, recent studies have incorporated time-dependent AUCs to evaluate model performance across various time points, offering a dynamic assessment of predictive accuracy in survival analysis (33). For hypertension, both GradientBoostingSurvival (mean AUC = 0.798) and CoxPHSurvival (mean AUC = 0.794) demonstrate strong predictive performance, closely followed by FastKernelSurvivalSVM (mean AUC = 0.791). For coronary heart disease, RandomSurvivalForest initially shows a highest AUC. For heart failure, CoxPHSurvival (mean AUC = 0.672) emerges as the top-performing model, with other models showing relatively lower AUCs, particularly in the early time points. For myocardial infarction, RandomSurvivalForest (mean AUC = 0.743) has a slight edge over the other models, though their AUCs remain close. Lastly, for stroke, CoxPHSurvival (mean AUC = 0.691) leads in performance, while the others exhibit lower and more similar AUCs, highlighting the complexity of predicting stroke mortality.

In recent years, the interpretability of machine learning models has seen significant growth, with visualization techniques playing a key role in enhancing the understanding of complex black-box models (27, 28, 34). Permutation feature importance analysis is widely used across various machine learning models to evaluate and rank the significance of individual features (26). Previous studies have utilized SHAP plot to identify key features in predicting coronary heart disease outcomes among individuals exposed to heavy metals (25). The application of SHAP explanations helps in providing a detailed understanding of the conditional effects of individual instances. In this context, positive SHAP values signify that certain feature values are associated with an increased risk of cardiovascular mortality over the 16-year NHANES survey, while negative SHAP values indicate a reduced mortality risk. In the present study, we developed five predictive models for cardiovascular diseases using a large sample from the NHANES database. To interpret these models, we applied three techniques: feature importance analysis, SHAP summary plots, and SHAP waterfall plots, offering a comprehensive insight into the relationship between heavy metal exposure and cardiovascular mortality. Our analysis identified the top five heavy metals most significantly associated with different cardiovascular diseases. For hypertension, the key metals are thallium, lead, and mercury. In coronary heart disease, the top three include cesium, thallium, and lead. Heart failure is primarily associated with cobalt, antimony, and cesium. For myocardial infarction, the important metals are lead, mercury, and cadmium. Lastly, stroke is most associated with cadmium, mercury, and lead. These differential associations highlight the value of metal-specific prevention strategies tailored to individual cardiovascular disease risks.

Lead emerged as a significant metal associated with multiple cardiovascular diseases in our analysis. Lead exposure, even at low concentrations, contributes to cardiovascular disease through multiple mechanisms (7, 35). It induces oxidative stress and systemic inflammation (36), damaging endothelial cells (37) and altering vascular reactivity (38). Simultaneously, lead disrupts lipid metabolism—elevating LDL and triglycerides while lowering HDL—thereby promoting a pro-atherosclerotic profile (5). Studies also suggest that lead accelerates telomere shortening, which in turn is linked to cellular senescence, impaired DNA repair, and heightened vascular aging (35). In addition, stored lead in bone can be mobilized over time, resulting in chronic, low-grade internal exposure that further exacerbates blood pressure elevation and vascular dysfunction (39). These combined processes—oxido-inflammatory damage, lipid disturbances, telomere attrition, and continued internal release—ultimately increase the risk of hypertension, atherosclerosis, and other cardiovascular pathologies, underscoring the importance of minimizing lead exposure at all levels.

Thallium also emerged as a critical heavy metal in our cardiovascular risk models. Thallium exposure, even at low doses, has been increasingly recognized as a risk factor for cardiovascular dysfunction through multiple biological pathways (35). Thallium closely mimics K^+^, enabling it to infiltrate neurons and myocardial cells readily, where it disrupts ionic homeostasis and mitochondrial function (40). Evidence suggests that thallium promotes oxidative stress – generating excess reactive oxygen species (ROS) – which, combined with potential interference in energy metabolism, damages vascular endothelial and cardiac cells (41, 42). Animal and human studies also indicate thallium can alter lipid metabolism, potentially increasing pro-atherogenic profiles (e.g., elevated LDL, decreased HDL) (43, 44). These combined insults – mitochondrial dysfunction, oxidative injury, and metabolic disturbances – create an environment conducive to hypertension, atherosclerosis, and other cardiovascular pathologies (45–47). Chronic low-level thallium exposure, including release from bone stores, amplifies these risks over time, underscoring the need to minimize Thallium contamination and rigorously monitor cardiovascular health among exposed populations (48). Similarly, mercury poses profound cardiovascular risks through heightened oxidative stress, endothelial dysfunction, and mitochondrial damage, which together escalate the likelihood of hypertension, atherosclerosis, and other vascular pathologies (49).

This study has several limitations. First, cardiovascular disease diagnoses were partly determined from participants’ self-reported information in the NHANES interview questionnaire, which may have led to information bias due to cognitive impairment or recall errors. Second, despite the strong performance of the survival models, further external validation with independent datasets is crucial to establishing their clinical utility and generalizability. Third, while our dataset contained sufficient cases of hypertension, the relatively smaller number of participants with other cardiovascular conditions (coronary heart disease, heart failure, myocardial infarction, and stroke) may have limited the statistical power for these analyses and potentially affected the robustness of our prediction models for these specific outcomes. This class imbalance could have resulted in models that perform well on majority cases but with reduced sensitivity for detecting relationships in the less prevalent conditions. Fourth, we were unable to differentiate between ischemic and hemorrhagic stroke in our analysis due to limitations in the NHANES dataset, which does not provide this level of diagnostic detail. This is an important limitation since these stroke subtypes have distinct pathophysiological mechanisms and potentially different relationships with heavy metal exposures. Fifth, there is a temporal mismatch between sample collection and disease diagnosis, as heavy metal concentrations were measured at the time of NHANES participation, which may not reflect the levels present when cardiovascular diseases were initially diagnosed. This cross-sectional nature of biomarker measurement means we cannot establish whether the observed metal concentrations preceded disease development or resulted from physiological changes due to the diseases themselves. Sixth, this is an observational study, and while we identified significant associations between specific heavy metals and cardiovascular outcomes, these findings cannot establish causality. Seventh, our analysis does not account for cardiovascular medications or interventions as covariates. This omission may have confounded our assessment of metal-cardiovascular relationships, as treatments substantially modify disease outcomes. Future research should incorporate treatment data to better isolate the independent effects of heavy metal exposures on cardiovascular mortality.

## Conclusion

In this study, we examined the association between heavy metal exposure and cardiovascular disease mortality among NHANES 2003–2018 participants using various machine learning models. For hypertension, GradientBoostingSurvival showed superior predictive capabilities. CoxPHSurvival demonstrated the most consistent performance in predicting mortality for heart failure and stroke, while RandomSurvivalForest was the top model for coronary heart disease and myocardial infarction. ExtraSurvivalTrees performed best in angina. Key metals for hypertension included thallium, lead, and mercury. For coronary heart disease, cesium, thallium, and lead were most relevant, while heart failure was linked to arsenic acid, cobalt, and antimony. Myocardial infarction was associated with lead, total mercury, and cadmium, and stroke was associated with cadmium, mercury, and lead. For angina, lead and cobalt are the key heavy metal. Recognizing these metals as key cardiovascular risk factors calls for stronger public health actions—like better surveillance and regulation—to reduce harmful exposures. Clinically, advanced survival models help doctors identify high-risk patients sooner and tailor treatments for better outcomes. Furthermore, we developed a user-friendly web calculator that allows clinicians and patients to compute individualized survival predictions for different cardiovascular diseases, offering a practical tool for risk assessment and informed decision-making.

## Data Availability

The original contributions presented in the study are included in the article/Supplementary material, further inquiries can be directed to the corresponding authors.

## Footnotes

^1^ https://www.cdc.gov/nchs/nhanes/Index.htm

## Supplementary material

SUPPLEMENTARY FIGURE S1 Flowchart of participant selection.

SUPPLEMENTARY FIGURE S2 Calibration plots of machine learning models for hypertension.

SUPPLEMENTARY FIGURE S3 Calibration plots of machine learning models for coronary heart disease.

SUPPLEMENTARY FIGURE S4 Calibration plots of machine learning models for heart failure.

SUPPLEMENTARY FIGURE S5 Calibration plots of machine learning models for myocardial infarction.

SUPPLEMENTARY FIGURE S6 Calibration plots of machine learning models for stroke.

SUPPLEMENTARY FIGURE S7 Calibration plots of machine learning models for angina.

SUPPLEMENTARY FIGURE S8 SHAP waterfall plots for individual patients. SHAP was used in personalized decision analysis to depict patient profiles for mortality prediction. The red bars indicate that the variable contributes to an increased mortality risk, whereas the blue bars suggest an inhibitory effect. **(A)** Hypertension: GradientBoostingSurvival, **(B)** coronary heart disease: RandomSurvivalForest, **(C)** heart failure: CoxPHSurvival, **(D)** myocardial infarction: RandomSurvivalForest, **(E)** stroke: CoxPHSurvival, **(F)** Angina: ExtraSurvivalTrees.

SUPPLEMENTARY FIGURE S9 SHAP decision plots illustrating the model’s decision-making in predicting cardiovascular disease mortality from a global perspective as variables change. The red line indicates a higher predicted mortality risk, while the blue line signifies a lower risk. **(A)** Hypertension: GradientBoostingSurvival, **(B)** coronary heart disease: RandomSurvivalForest, **(C)** heart failure: CoxPHSurvival, **(D)** myocardial infarction: RandomSurvivalForest, **(E)** stroke: CoxPHSurvival, **(F)** Angina: ExtraSurvivalTrees.

## References

[1] WHO. The top 10 causes of death. (2024). Available at: https://www.who.int/news-room/fact-sheets/detail/the-top-10-causes-of-death

[2] Shameer K Johnson KW Glicksberg BS Dudley JT Sengupta PP. Machine learning in cardiovascular medicine: are we there yet? Heart. (2018) 104:1156–64. doi: 10.1136/heartjnl-2017-311198 29352006

[3] Nurmohamed NS van Rosendael AR Danad I Ngo-Metzger Q Taub PR Ray KK. Atherosclerosis evaluation and cardiovascular risk estimation using coronary computed tomography angiography. Eur Heart J. (2024) 45:1783–800. doi: 10.1093/eurheartj/ehae190, PMID: 38606889 PMC11129796

[4] Johnson KW Torres Soto J Glicksberg BS Shameer K Miotto R Ali M. Artificial Intelligence in Cardiology. J Am Coll Cardiol. (2018) 71:2668–79. doi: 10.1016/j.jacc.2018.03.521, PMID: 29880128

[5] Pan Z Gong T Liang P. Heavy metal exposure and cardiovascular disease. Circ Res. (2024) 134:1160–78. doi: 10.1161/circresaha.123.323617, PMID: 38662861

[6] Verzelloni P Urbano T Wise LA Vinceti M Filippini T. Cadmium exposure and cardiovascular disease risk: a systematic review and dose-response meta-analysis. Environ Pollut. (2024) 345:123462. doi: 10.1016/j.envpol.2024.123462, PMID: 38295933

[7] Navas-Acien A Guallar E Silbergeld EK Rothenberg SJ. Lead exposure and cardiovascular disease--a systematic review. Environ Health Perspect. (2007) 115:472–82. doi: 10.1289/ehp.9785, PMID: 17431501 PMC1849948

[8] Lanphear BP Rauch S Auinger P Allen RW Hornung RW. Low-level lead exposure and mortality in US adults: a population-based cohort study. Lancet Public Health. (2018) 3:e177–84. doi: 10.1016/s2468-2667(18)30025-2, PMID: 29544878

[9] Tellez-Plaza M Navas-Acien A Menke A Crainiceanu CM Pastor-Barriuso R Guallar E. Cadmium exposure and all-cause and cardiovascular mortality in the U.S. general population. Environ Health Perspect. (2012) 120:1017–22. doi: 10.1289/ehp.1104352, PMID: 22472185 PMC3404657

[10] Jomova K Alomar SY Nepovimova E Kuca K Valko M. Heavy metals: toxicity and human health effects. Arch Toxicol. (2025) 99:153–209. doi: 10.1007/s00204-024-03903-2, PMID: 39567405 PMC11742009

[11] Wang X Mukherjee B Park SK. Associations of cumulative exposure to heavy metal mixtures with obesity and its comorbidities among U.S. adults in NHANES 2003-2014. Environ Int. (2018) 121:683–94. doi: 10.1016/j.envint.2018.09.035, PMID: 30316184 PMC6268112

[12] Fagerberg B Kjelldahl J Sallsten G Barregard L Forsgard N Österberg K. Cadmium exposure as measured in blood in relation to macrophage density in symptomatic atherosclerotic plaques from human carotid artery. Atherosclerosis. (2016) 249:209–14. doi: 10.1016/j.atherosclerosis.2016.01.011, PMID: 27156912

[13] Lin CH Hsu YT Yen CC Chen HH Tseng CJ Lo YK. Association between heavy metal levels and acute ischemic stroke. J Biomed Sci. (2018) 25:49. doi: 10.1186/s12929-018-0446-0, PMID: 29801491 PMC5970463

[14] Leone N Courbon D Ducimetiere P Zureik M. Zinc, copper, and magnesium and risks for all-cause, cancer, and cardiovascular mortality. Epidemiology. (2006) 17:308–14. doi: 10.1097/01.ede.0000209454.41466.b7, PMID: 16570028

[15] Heo J Yoon JG Park H Kim YD Nam HS Heo JH. Machine learning-based model for prediction of outcomes in acute stroke. Stroke. (2019) 50:1263–5. doi: 10.1161/strokeaha.118.024293, PMID: 30890116

[16] Li J Liu S Hu Y Zhu L Mao Y Liu J. Predicting mortality in intensive care unit patients with heart failure using an interpretable machine learning model: retrospective cohort study. J Med Internet Res. (2022) 24:e38082. doi: 10.2196/38082, PMID: 35943767 PMC9399880

[17] Forrest IS Petrazzini BO Duffy Á Park JK Marquez-Luna C Jordan DM. Machine learning-based marker for coronary artery disease: derivation and validation in two longitudinal cohorts. Lancet. (2023) 401:215–25. doi: 10.1016/s0140-6736(22)02079-7, PMID: 36563696 PMC10069625

[18] Doudesis D Lee KK Boeddinghaus J Bularga A Ferry AV Tuck C. Machine learning for diagnosis of myocardial infarction using cardiac troponin concentrations. Nat Med. (2023) 29:1201–10. doi: 10.1038/s41591-023-02325-4, PMID: 37169863 PMC10202804

[19] Ambale-Venkatesh B Yang X Wu CO Liu K Hundley WG McClelland R. Cardiovascular event prediction by machine learning: the multi-ethnic study of atherosclerosis. Circ Res. (2017) 121:1092–101. doi: 10.1161/circresaha.117.311312, PMID: 28794054 PMC5640485

[20] Heagerty PJ Lumley T Pepe MS. Time-dependent ROC curves for censored survival data and a diagnostic marker. Biometrics. (2000) 56:337–44. doi: 10.1111/j.0006-341x.2000.00337.x 10877287

[21] Bhatnagar A. Environmental cardiology: studying mechanistic links between pollution and heart disease. Circ Res. (2006) 99:692–705. doi: 10.1161/01.RES.0000243586.99701.cf, PMID: 17008598

[22] Li X Zhang D Zhao Y Kuang L Huang H Chen W. Correlation of heavy metals' exposure with the prevalence of coronary heart disease among US adults: findings of the US NHANES from 2003 to 2018. Environ Geochem Health. (2023) 45:6745–59. doi: 10.1007/s10653-023-01670-0 37378736

[23] Mandal R Kaur S Gupta VK Joshi A. Heavy metals controlling cardiovascular diseases risk factors in myocardial infarction patients in critically environmentally heavy metal-polluted steel industrial town Mandi-Gobindgarh (India). Environ Geochem Health. (2022) 44:3215–38. doi: 10.1007/s10653-021-01068-w, PMID: 34455537

[24] Xing X Xu M Yang L Shao C Wang Y Qi M. Association of selenium and cadmium with heart failure and mortality based on the National Health and nutrition examination survey. J Hum Nutr Diet. (2023) 36:1496–506. doi: 10.1111/jhn.13107, PMID: 36321401

[25] Li X Zhao Y Zhang D Kuang L Huang H Chen W. Development of an interpretable machine learning model associated with heavy metals' exposure to identify coronary heart disease among US adults via SHAP: findings of the US NHANES from 2003 to 2018. Chemosphere. (2023) 311:137039. doi: 10.1016/j.chemosphere.2022.137039 36342026

[26] Li W Huang G Tang N Lu P Jiang L Lv J. Effects of heavy metal exposure on hypertension: a machine learning modeling approach. Chemosphere. (2023) 337:139435. doi: 10.1016/j.chemosphere.2023.139435 37422210

[27] Yao J Du Z Yang F Duan R Feng T. The relationship between heavy metals and metabolic syndrome using machine learning. Front Public Health. (2024) 12:1378041. doi: 10.3389/fpubh.2024.1378041, PMID: 38686033 PMC11057329

[28] Gui Y Gui S Wang X Li Y Xu Y Zhang J. Exploring the relationship between heavy metals and diabetic retinopathy: a machine learning modeling approach. Sci Rep. (2024) 14:13049. doi: 10.1038/s41598-024-63916-w, PMID: 38844504 PMC11156935

[29] Xia F Li Q Luo X Wu J. Machine learning model for depression based on heavy metals among aging people: a study with National Health and nutrition examination survey 2017-2018. Front Public Health. (2022) 10:939758. doi: 10.3389/fpubh.2022.939758, PMID: 35991018 PMC9386350

[30] Xia F Li Q Luo X Wu J. Identification for heavy metals exposure on osteoarthritis among aging people and machine learning for prediction: a study based on NHANES 2011-2020. Front Public Health. (2022) 10:906774. doi: 10.3389/fpubh.2022.906774, PMID: 35979456 PMC9376265

[31] Duan W Xu C Liu Q Xu J Weng Z Zhang X. Levels of a mixture of heavy metals in blood and urine and all-cause, cardiovascular disease and cancer mortality: a population-based cohort study. Environ Pollut. (2020) 263:114630. doi: 10.1016/j.envpol.2020.114630, PMID: 33618481

[32] Khera R Haimovich J Hurley NC McNamara R Spertus JA Desai N. Use of machine learning models to predict death after acute myocardial infarction. JAMA Cardiol. (2021) 6:633–41. doi: 10.1001/jamacardio.2021.0122, PMID: 33688915 PMC7948114

[33] Smith AH Gray GM Ashfaq A Asante-Korang A Rehman MA Ahumada LM. Using machine learning to predict five-year transplant-free survival among infants with hypoplastic left heart syndrome. Sci Rep. (2024) 14:4512. doi: 10.1038/s41598-024-55285-1, PMID: 38402363 PMC10894293

[34] Chen H Wang M Li J. Exploring the association between two groups of metals with potentially opposing renal effects and renal function in middle-aged and older adults: evidence from an explainable machine learning method. Ecotoxicol Environ Saf. (2024) 269:115812. doi: 10.1016/j.ecoenv.2023.115812, PMID: 38091680

[35] He L Chen Z Dai B Li G Zhu G. Low-level lead exposure and cardiovascular disease: the roles of telomere shortening and lipid disturbance. J Toxicol Sci. (2018) 43:623–30. doi: 10.2131/jts.43.623, PMID: 30404996

[36] Obeng-Gyasi E Obeng-Gyasi B. Association of combined lead, cadmium, and mercury with systemic inflammation. Front Public Health. (2024) 12:1385500. doi: 10.3389/fpubh.2024.1385500, PMID: 39267632 PMC11390544

[37] Hara T Kumagai R Tanaka T Nakano T Fujie T Fujiwara Y. Lead suppresses perlecan expression via EGFR-ERK1/2-COX-2-PGI(2) pathway in cultured bovine vascular endothelial cells. J Toxicol Sci. (2023) 48:655–63. doi: 10.2131/jts.48.655, PMID: 38044127

[38] Xie X Wang K Shen X li X Wang S Yuan S. Potential mechanisms of aortic medial degeneration promoted by co-exposure to microplastics and lead. J Hazard Mater. (2024) 475:134854. doi: 10.1016/j.jhazmat.2024.134854, PMID: 38889468

[39] Barry V Todd AC Steenland K. Bone lead associations with blood lead, kidney function and blood pressure among US, lead-exposed workers in a surveillance programme. Occup Environ Med. (2019) 76:349–54. doi: 10.1136/oemed-2018-105505, PMID: 30661026

[40] Nagel A Cuss CW Goss GG Shotyk W Glover CN. Mechanistic examination of thallium and potassium interactions in *Daphnia magna*. Comp Biochem Physiol C Toxicol Pharmacol. (2023) 271:109686. doi: 10.1016/j.cbpc.2023.109686, PMID: 37343692

[41] Korotkov SM. Mitochondrial oxidative stress is the general reason for apoptosis induced by different-valence heavy metals in cells and mitochondria. Int J Mol Sci. (2023) 24:14459. doi: 10.3390/ijms241914459, PMID: 37833908 PMC10572412

[42] Chen X Li P Huang Y Lv Y Xu X Nong H. Joint associations among non-essential heavy metal mixtures and nutritional factors on glucose metabolism indexes in US adults: evidence from the NHANES 2011-2016. Food Funct. (2024) 15:2706–18. doi: 10.1039/d3fo05439j, PMID: 38376466

[43] Li D Yao H Zhu X Li Z Zeng X. Thallium(I) exposure perturbs the gut microbiota and metabolic profile as well as the regional immune function of C57BL/6 J mice. Environ Sci Pollut Res Int. (2022) 29:90495–508. doi: 10.1007/s11356-022-22145-2, PMID: 35870064

[44] Galván-Arzate S Pedraza-Chaverrí J Medina-Campos ON Maldonado PD Vázquez-Román B Ríos C. Delayed effects of thallium in the rat brain: regional changes in lipid peroxidation and behavioral markers, but moderate alterations in antioxidants, after a single administration. Food Chem Toxicol. (2005) 43:1037–45. doi: 10.1016/j.fct.2005.02.006, PMID: 15833379

[45] Lin G Sun Y Long J Sui X Yang J Wang Q. Involvement of the Nrf2-Keap1 signaling pathway in protection against thallium-induced oxidative stress and mitochondrial dysfunction in primary hippocampal neurons. Toxicol Lett. (2020) 319:66–73. doi: 10.1016/j.toxlet.2019.11.008 31726083

[46] Chen CS Yuan TH Lu TP Lee HY Chen YH Lai LC. Exposure-associated DNA methylation among people exposed to multiple industrial pollutants. Clin Epigenetics. (2024) 16:111. doi: 10.1186/s13148-024-01705-y, PMID: 39164771 PMC11337639

[47] Chang HF Tseng SC Tang MT Hsiao SSY Lee DC Wang SL. Physiology and molecular basis of thallium toxicity and accumulation in *Arabidopsis thaliana*. Ecotoxicol Environ Saf. (2024) 276:116290. doi: 10.1016/j.ecoenv.2024.116290, PMID: 38599154

[48] Liu J Wang K. Disentangling the relationship between urinary metal exposure and osteoporosis risk across a broad population: a comprehensive supervised and unsupervised analysis. Toxics. (2024) 12:866. doi: 10.3390/toxics12120866, PMID: 39771081 PMC11679131

[49] Houston MC. Role of mercury toxicity in hypertension, cardiovascular disease, and stroke. J Clin Hypertens (Greenwich). (2011) 13:621–7. doi: 10.1111/j.1751-7176.2011.00489.x, PMID: 21806773 PMC8108748

