# Supplementary figures and images for: Developing machine learning models for predicting cardiovascular disease survival based on heavy metal serum and urine levels
Source: Front Public Health. 2025 May 21;13:1582779. doi: 10.3389/fpubh.2025.1582779 (PMC12134072; doi:10.3389/fpubh.2025.1582779)

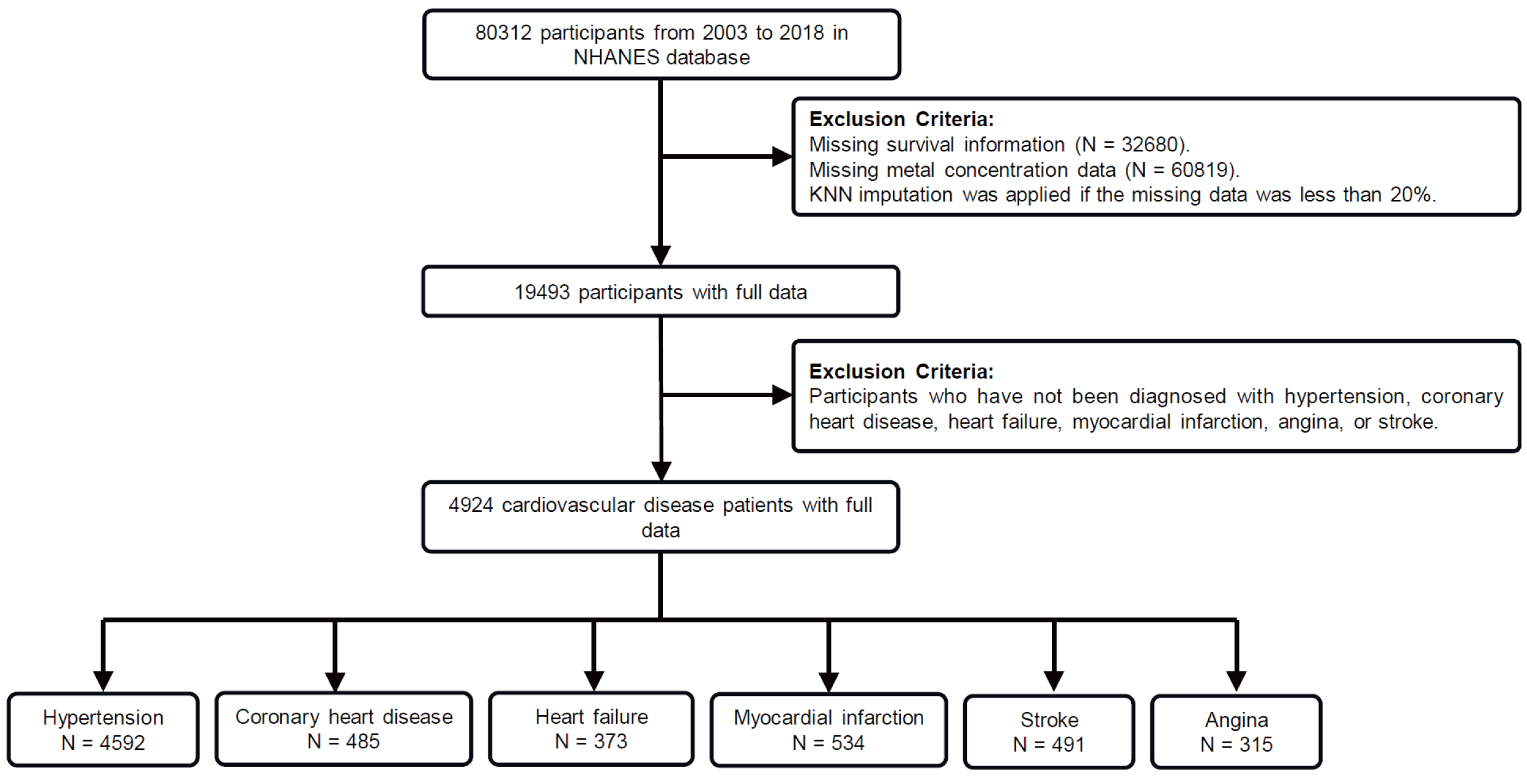

Supplement: SUPPLEMENTARY FIGURE S1 — Flowchart of participant selection. [file Image_1.tif]

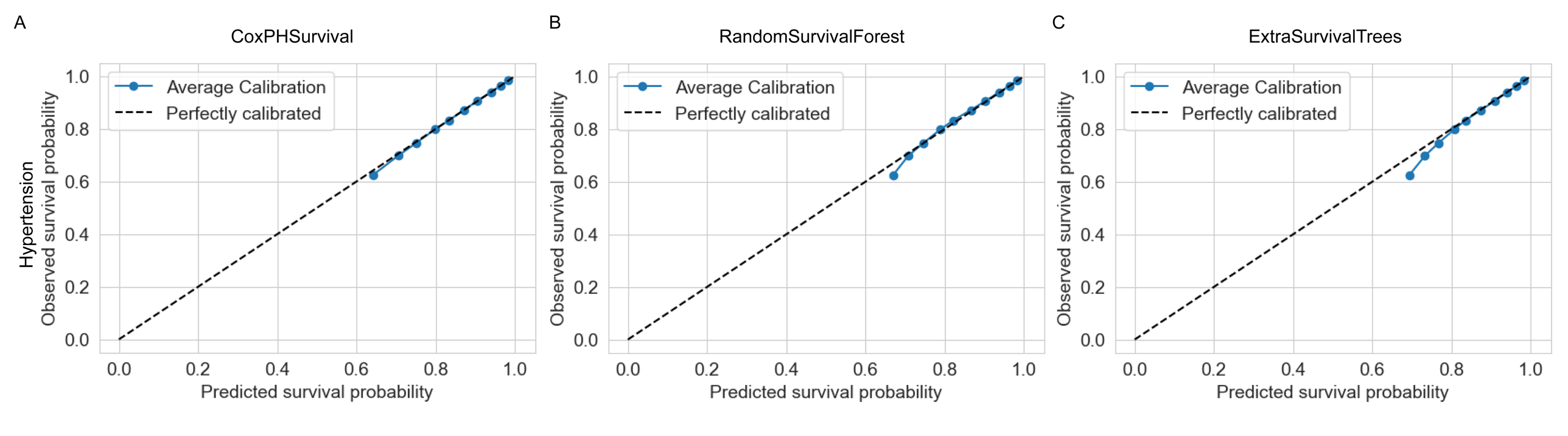

Supplement: SUPPLEMENTARY FIGURE S2 — Calibration plots of machine learning models for hypertension. [file Image_2.tif]

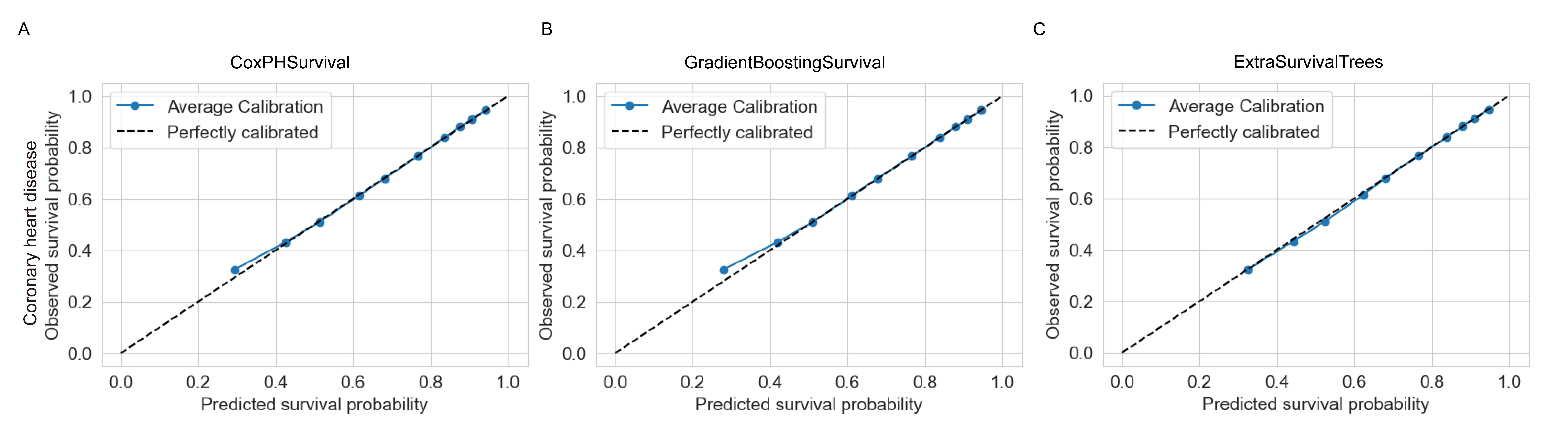

Supplement: SUPPLEMENTARY FIGURE S3 — Calibration plots of machine learning models for coronary heart disease. [file Image_3.tif]

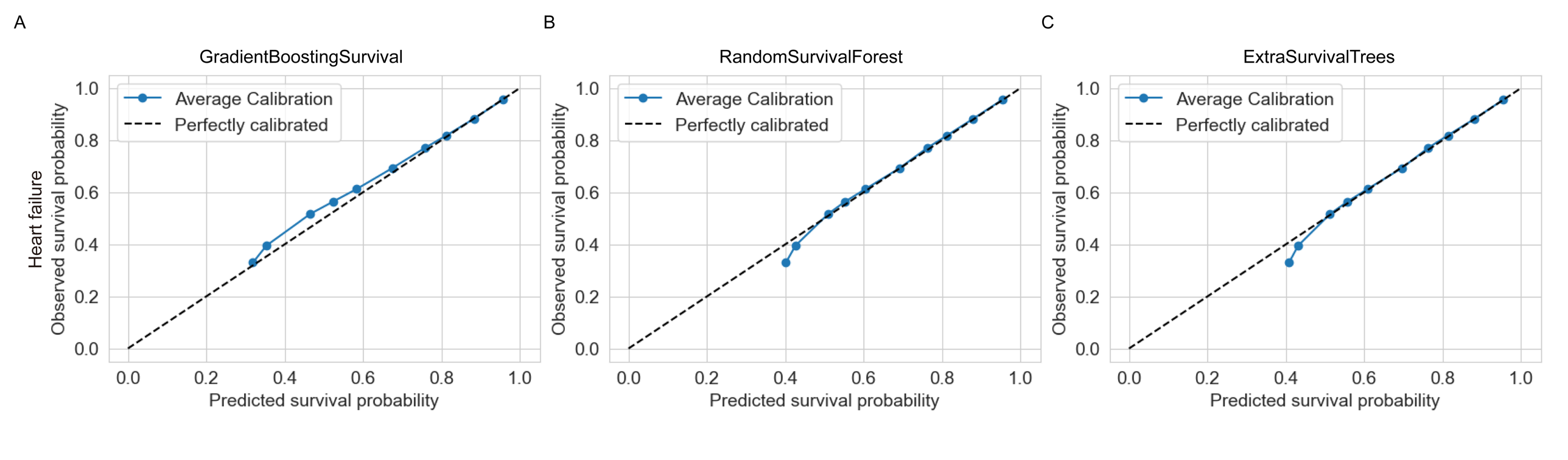

Supplement: SUPPLEMENTARY FIGURE S4 — Calibration plots of machine learning models for heart failure. [file Image_4.tif]

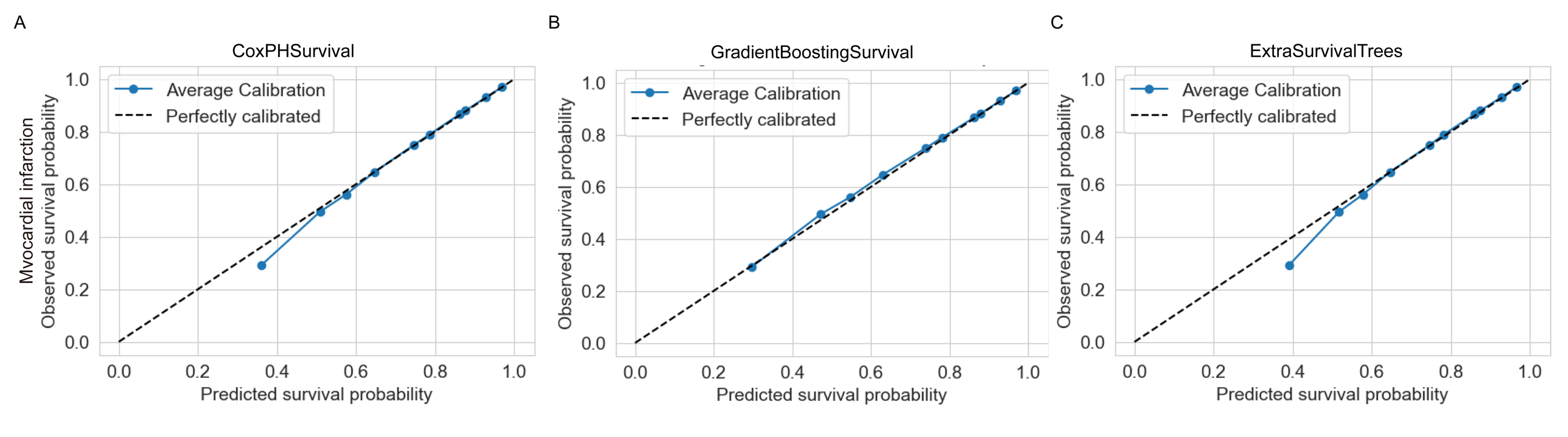

Supplement: SUPPLEMENTARY FIGURE S5 — Calibration plots of machine learning models for myocardial infarction. [file Image_5.tif]

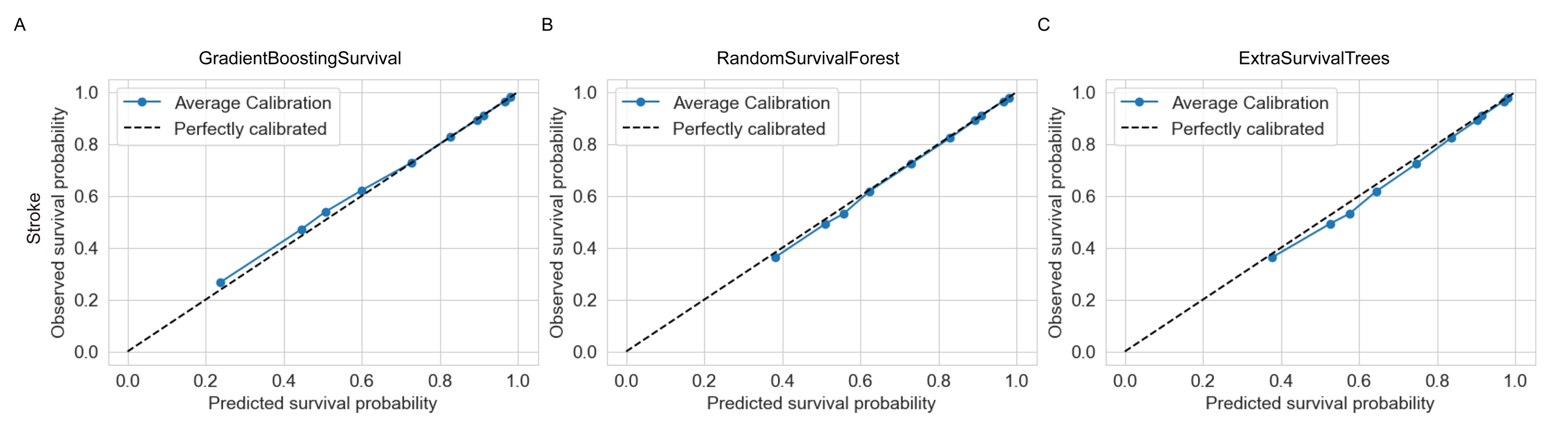

Supplement: SUPPLEMENTARY FIGURE S6 — Calibration plots of machine learning models for stroke. [file Image_6.tif]

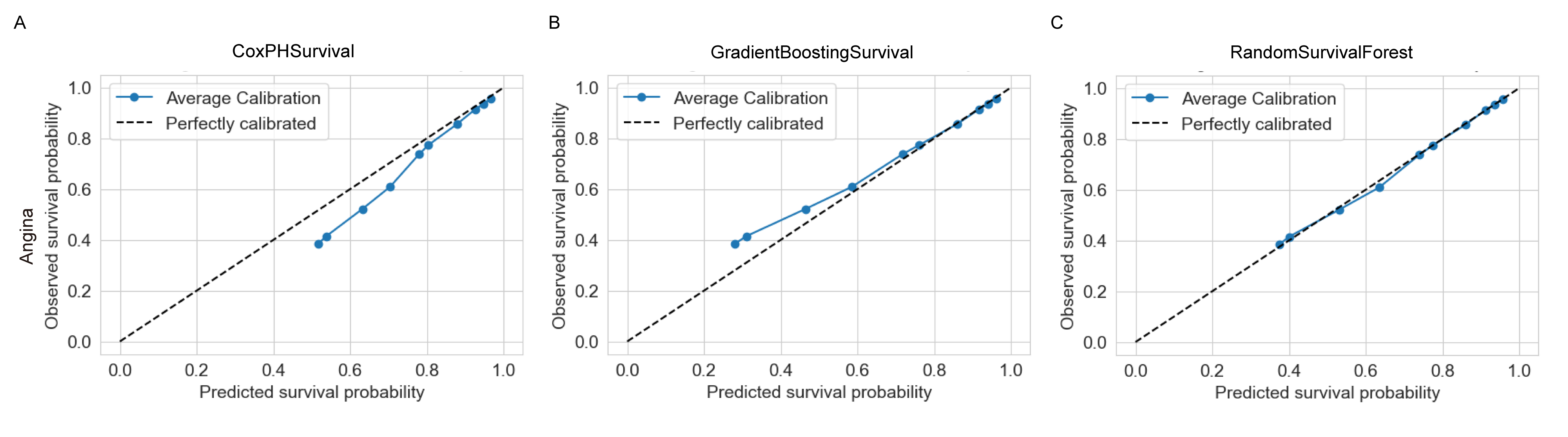

Supplement: SUPPLEMENTARY FIGURE S7 — Calibration plots of machine learning models for angina. [file Image_7.tif]

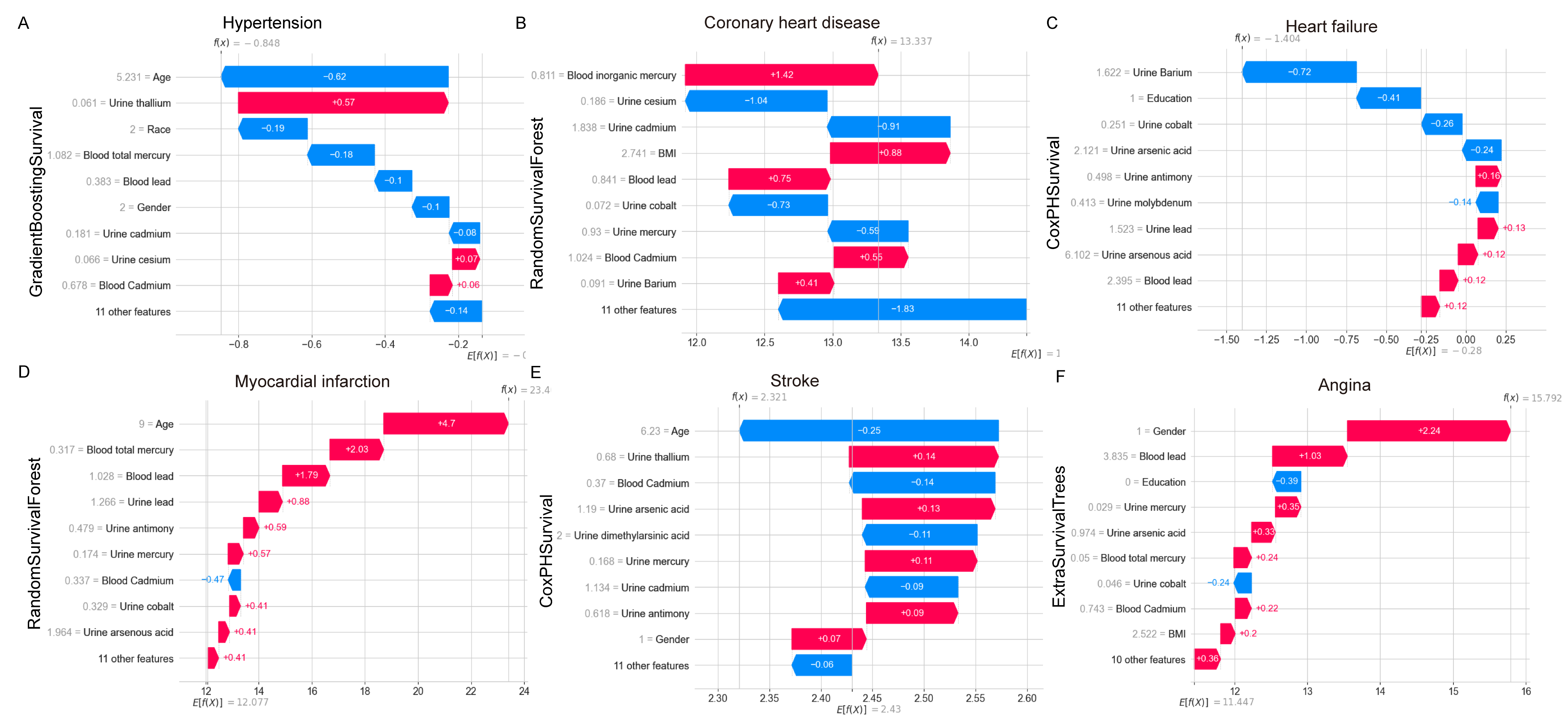

Supplement: SUPPLEMENTARY FIGURE S8 — SHAP waterfall plots for individual patients. SHAP was used in personalized decision analysis to depict patient profiles for mortality prediction. The red bars indicate that the variable contributes to an increased mortality risk, whereas the blue bars suggest an inhibitory effect. (A) Hypertension: GradientBoostingSurvival, (B) coronary heart disease: RandomSurvivalForest, (C) heart failure: CoxPHSurvival, (D) myocardial infarction: RandomSurvivalForest, (E) stroke: CoxPHSurvival, (F) Angina: ExtraSurvivalTrees. [file Image_8.tif]

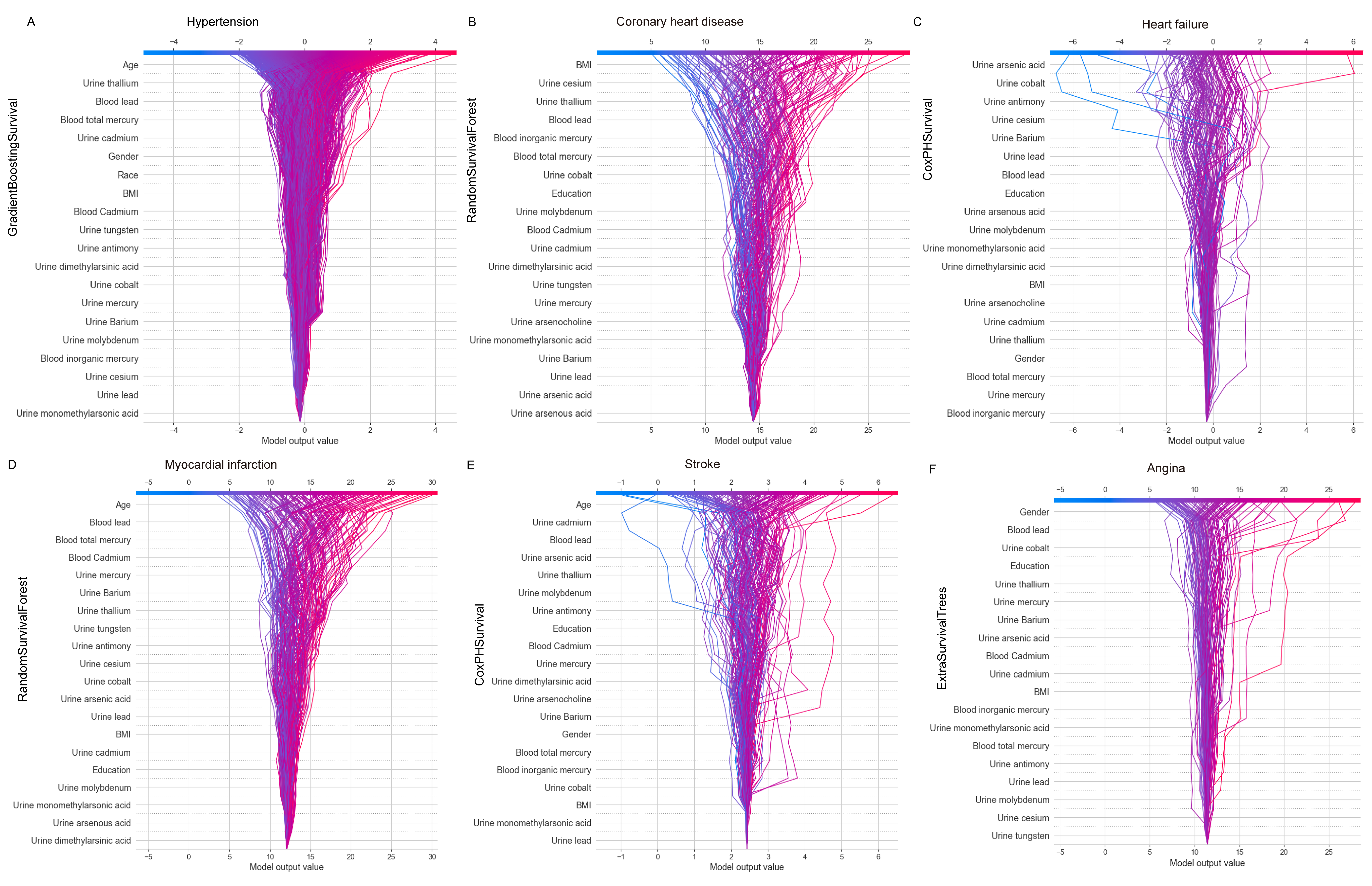

Supplement: SUPPLEMENTARY FIGURE S9 — SHAP decision plots illustrating the model’s decision-making in predicting cardiovascular disease mortality from a global perspective as variables change. The red line indicates a higher predicted mortality risk, while the blue line signifies a lower risk. (A) Hypertension: GradientBoostingSurvival, (B) coronary heart disease: RandomSurvivalForest, (C) heart failure: CoxPHSurvival, (D) myocardial infarction: RandomSurvivalForest, (E) stroke: CoxPHSurvival, (F) Angina: ExtraSurvivalTrees. [file Image_9.tif]
